# The Acute Effects of Cold Water Immersion and Percussive Massage Therapy on Neuromuscular Properties and Muscle Soreness after Exercise in Young Male Soccer Players

**DOI:** 10.3390/sports12060167

**Published:** 2024-06-15

**Authors:** Alex Buoite Stella, Angelo Michele Dragonetti, Simone Fontanot, Raffaele Sabot, Miriam Martini, Alessandra Galmonte, Gianluca Canton, Manuela Deodato, Luigi Murena

**Affiliations:** 1 School of Physiotherapy, Department of Medicine, Surgery and Health Sciences, University of Trieste, Via Pascoli 31, 34100 Trieste, Italy; abuoitestella@units.it (A.B.S.); miriam.martini@phd.units.it (M.M.); agalmonte@units.it (A.G.); gcanton@units.it (G.C.); mdeodato@units.it (M.D.); lmurena@units.it (L.M.); 2 PhD Program in Personalized Medicine and Innovative Therapies, Department of Medicine, Surgery and Health Sciences, University of Trieste, 34100 Trieste, Italy; 3 Orthopedics and Traumatology Unit, Department of Medicine, Surgery and Health Sciences, University of Trieste, Strada di Fiume 447, 34149 Trieste, Italy

**Keywords:** recovery, team sport, cryotherapy, massage guns

## Abstract

Cold water immersion (CWI) and percussive massage therapy (PMT) are commonly used recovery techniques in team sports. In particular, despite its wide use, PMT has been scarcely investigated in the literature, especially regarding neuromuscular measures and in comparison with other techniques. This study aimed to evaluate and compare the acute and short-term effects (24 h) of CWI and PMT on muscle strength, contractile properties, and soreness after exercise. A randomized crossover study was performed on sixteen male soccer players (22 years, 20–27) who participated in three experimental sessions involving a fatiguing protocol consisting of a Yo-Yo Intermittent Endurance Test followed by 3 × 10 squat jumps and a wall sit for 30 s, and 12 min of recovery including CWI (10 °C water), bilateral PMT on the anterior and posterior thigh, or passive resting. Outcomes were assessed immediately after the exercise protocol, after the recovery intervention, and at 24 h. Isometric knee extension (IKE) and flexion (IKF) and tensiomyography (TMG) were assessed. Muscle soreness and fatigue were scored from 0 to 10. PMT increased strength after the treatment (*p* = 0.004) and at 24 h (*p* = 0.007), whereas no significant differences were found for the other two recovery modalities. At post-recovery, compared to CON, CWI resulted in a longer TMG contraction time (*p* = 0.027). No significant differences were found at 24 h. Finally, PMT and CWI enhanced muscle soreness recovery compared to passive rest (F_4,60_ = 3.095, *p* = 0.022, _p_η^2^ = 0.171). Preliminary results from this study suggest that PMT might improve isometric strength after strenuous exercise, and both PMT and CWI reduce muscle soreness perception, while the effects on TMG parameters remain controversial.

## 1. Introduction

Proper recovery after training or competition has been suggested to influence subsequent performance and should be recommended to reduce the risk of overreaching or other sport-related injuries [1]. In particular, recovery strategies could be helpful in mitigating the effects of fatigue and pain on exercise capacity, allowing higher loads to be tolerated and sport-specific performance to be improved [1]. According to the scientific literature, several strategies and techniques can be used to accelerate recovery, such as nutrition/hydration, cold/hot water immersion, sleeping, active recovery, stretching, compression garments, massage, and electrical stimulation [2]. 

Cold water immersion (CWI) represents one of the most used recovery techniques, especially in team sports and soccer, with athletes considering CWI one of the most effective treatments for recovery purposes [3]. Despite the exact mechanisms underlying the effects of CWI on fatigue and recovery remaining elusive [4], some research suggests that it might attenuate the effects of strenuous exercise, acting as an analgesic to reduce muscle pain, swelling, and post-exercise inflammation [4]. As such, some authors found that it could be useful in improving physical performance [5], and despite some conflicting results [4,6], it is usually well accepted as a low-effort approach, thanks also to the development of different portable tubs and systems [1]. Nevertheless, CWI often lacks standardized protocols in terms of water temperature, timing, and duration of the immersion, and a possible explanation for the inconsistent findings could depend on interindividual differences [7]. Among other passive recovery strategies, percussive devices have recently become popular among athletes as self-treatment options by combining compression and vibration [8]. In particular, the scientific rationale underlying the development of such devices includes the effects of compression and vibration on blood flow, inflammation, muscle soreness and stiffness, and fatigue perception [8,9]. To date, only a few studies have evaluated the effects of percussive massage treatment (PMT) on recovery after exercise, suggesting a transient improvement in joint range of motion and reduced muscle stiffness, with conflicting results on muscle performance [10,11], and the influence of percussive massage on the biomechanical parameters of muscles has been suggested, but without affecting muscle strength [12]. Such devices can operate with different protocols in terms of amplitude, frequency, and type of attachment, according to manufacturer instructions based on the body area and aim; frequency has been the most commonly reported parameter, typically ranging from 30 to 53 Hz on the muscles of the lower limbs [10]. Soccer, like other team sports, is characterized by a high workload, including frequent training and the common possibility of playing additional games during the week [1]. Indeed, it has been reported that elite soccer players usually perform one game every 4.3 days, resulting in players participating in two games per week and fewer than or equal to four recovery days under substantial risk of sustaining an injury [13]. Since a 72 h post-match period might be not sufficient to completely restore the homeostatic balance, it becomes fundamental to accelerate recovery to improve performance and reduce the risk of injuries or illnesses [14]. 

Since the popularity of CWI and PMT among soccer players, it is expected that they might improve neuromuscular parameters compared to a passive recovery strategy (CON) in previously fatigued muscles by improving isometric strength and reducing muscle stiffness and soreness. As such, this study aimed to investigate the acute and short-term (24 h) effects of CWI, PMT, and CON on the muscle strength and contractile properties of the lower limbs, as well as the subjective measures of soreness and fatigue, after strenuous exercise in soccer players.

## 2. Materials and Methods

The present cross-over randomized study was performed between May and July 2023. This study was approved by the local university ethics committee (122/2022) and was conducted according to the Declaration of Helsinki principles. A signed informed consent was obtained from all subjects involved in this study.

Participants were recruited among local soccer clubs, with the following inclusion criteria: healthy males, aged between 16 and 30 years, training in soccer for more than 3 years, and with a training frequency > 2 times/week. According to McKay’s classification [15], included participants were in the Tier 2: Trained/Developmental group. Participants were excluded if, at the time of the first visit, they reported time-loss injuries, which are typically defined as injuries that restrict the athlete’s participation for at least 24 h beyond the report of injury, during the three months before the measurements, or if they suffered from injuries or illnesses that required a return to play >7 days in the previous year [16]. Participants were also excluded if they reported using analgesics or other therapies affecting muscle function or pain. During the period of this study, they were requested to refrain from training/competitions and from the use of protocols and devices that might affect the results, such as massage, compression garments, or electrical stimulation. 

**Figure 1:**
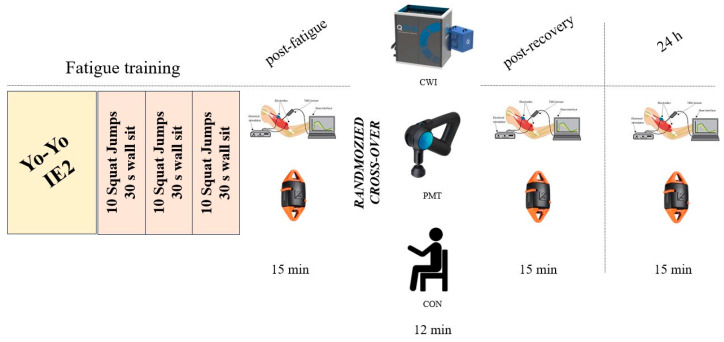
A schematic representation of the cross-over protocol showing the fatiguing protocol followed by the outcomes assessment (tensiomyography and isometric strength) and the recovery interventions (cold water immersion, CWI; percussive massage therapy, PMT; and passive rest, CON).

After the recruitment, all participants completed a demographics and anamnestic questionnaire, and exclusion criteria were evaluated by two independent physiotherapists according to the International Olympic Committee (IOC) consensus [16]. All the participants were familiarized with the outcome assessment procedures, and particularly the isometric knee extension (IKE) and isometric knee flexion (IKF) tests. Then, the participants were invited to participate in a 3-week cross-over study, including 3 measurement sessions separated by 1 week of rest (Figure 1). 

Every session included a fixed standardized fatiguing protocol and a randomized recovery intervention. The sequence of recovery intervention was randomized by a researcher not involved in the outcomes assessment, by drawing a card identifying the order of the interventions. The recovery-associated outcomes were assessed within 15 min from the ending of the fatiguing protocol (post fatigue), within 15 min after the recovery intervention (post recovery), and at 24 h. All the participants were tested at the same time of the day, in the afternoon. Participants were asked to avoid any strenuous physical activity in the 24 h before the measurements, and to refrain from food, smoking, coffee, and energy drinks in the 2 h before the fatiguing protocol. To reduce the influence of hydration status on the assessed outcomes [17], all the participants were instructed to consume 500 mL of plain water 30 min prior to the fatiguing protocol, and they were allowed to consume plain water ad libitum during all the experimental sessions. Before the fatiguing protocol, a 10 min warm-up was allowed and encouraged.

The Yo-Yo Intermittent Endurance Test—Level 2 (Yo-Yo IE2) was used as a standardized and sport-specific fatiguing protocol, consisting of 2 × 20 m repeated runs between 2 lines marked with cones with constantly increasing speed governed by signals from a smartphone speaker. Between each run, 5 s of active rest, light jogging on the spot, was given. When the test subject failed to reach the line twice, the test was considered completed. The Yo-Yo IE2 has been tested for validity and reliability and has previously been used as a fatiguing protocol in soccer players [18]. To further provide a fatiguing stimulus, after the Yo-Yo IE2, the participants were also asked to perform 3 sets without recovery, consisting of 10 squat jumps and a wall sit for 30 s.

### 2.1. Recovery Interventions

The control condition (PAS) consisted of participants resting on a chair for 12 min in a quiet room at 25 °C. Two weeks before the first experimental session, all the participants familiarized themselves with the proposed recovery interventions and outcomes assessment.

#### 2.1.1. CWI

The CWI protocol consisted of participants standing, with only their underwear and a t-shirt, in a portable tub for cold water therapy (Qryo, Italy), with the water to their hips being constantly maintained at 10 ± 0.5 °C by a cooling system that pumped and stirred the water inside the tub and around the participant’s lower limbs. Water temperature and the duration of the immersion, which was 12 min for all the participants considering they were characterized by similar anthropometrics and body composition, were based on the literature [7]. 

#### 2.1.2. PMT

The PMT protocol was performed with the application of a percussive therapy gun (Theragun Elite, Therabody, Orange, CA, USA). The participants rested with their underwear and a t-shirt on a treatment bed, and the percussive therapy gun with a “standard ball”, a 16 mm amplitude of percussions, and a frequency of 30 Hz was applied to the thigh muscles of both lower limbs, 3 min on the anterior and 3 min on the posterior area of each thigh. The protocol was based on the literature and adapted to the specific aims of this study [10]. 

### 2.2. Outcomes

#### 2.2.1. Isometric Muscle Strength

Isometric knee extension (IKE) and flexion (IKF) strength assessments were performed on a treatment bed with a digital handheld dynamometer (K-Pull, Kinvent, Montpellier, France) [19], which showed good validity and reliability [20]. For IKE, the participant was sitting on the border of the bed, in a standardized position of 90° hip flexion with a thigh block, and lumbar support was offered. For the IKF, the participant lay prone on the testing bed. The limb was assessed with the knee in 90° of flexion. The axis of movement in the knee, the lateral femoral condyle, was aligned with the axis of rotation of the dynamometer. All starting positions were checked with a goniometer and the dynamometer was aligned to be perpendicular to the leg. Two repetitions of each strength test were performed with a rest period of five seconds between each repetition and thirty seconds between each test. Participants were asked to gradually build up to a maximum force and maintain the effort, and then relax when instructed after a total of five seconds. Verbal encouragement was provided during testing to produce a maximum effort matched by the tester (make test) according to the literature [21]. The maximum value (kg) was chosen. No normalization was needed, since a paired-sample comparison was performed.

#### 2.2.2. Tensiomyography

Tensiomyography (TMG) is a validated technique to measure in vivo skeletal muscle mechanical contractile properties as a radial muscle belly displacement after electrical stimulation. TMG has been suggested to reflect skeletal muscle changes after training or immobilization, injuries and musculoskeletal illnesses, lateral and functional asymmetries, and altered parameters, which might reflect muscle damage and neuromuscular fatigue [19,22]. The TMG assessment included a single, 1 ms maximal monophasic electrical impulse used to elicit a twitch contraction that caused the muscle belly to oscillate. These oscillations were recorded using a sensitive digital displacement sensor (TMG-BMC Ltd., Ljubljana, Slovenia) placed on the skin’s surface at the measuring site of the muscle of interest. Initially, the stimulation amplitude was set just above the threshold and then gradually increased until the amplitude of the radial twitch (in millimeters) increased no further. Electrical pulses ranged between 85 and 110 mA at a constant 30 V. An inter-stimulation time interval of 10–15 s was used. From two maximal responses, all contractile parameters were estimated and average values were taken for further consideration. For this study, only Dm [the maximal displacement (mm)] and Tc [contraction time; the time between 10% and 90% of Dm (ms)] were taken into consideration and were extracted by TMG software (Version 3.6.16) and used for offline analysis, as they have been suggested to reflect muscle damage and fatigue [19]. Indeed, Dm is the absolute spatial transverse deformation of the muscle and a reduced Dm is interpreted as an increase in muscle stiffness, whereas a larger Dm implies lower muscle stiffness; Tc reflects the speed of twitch force generation, possibly reflecting muscle fiber type or tendon stiffness [22]. Participants were asked to lay on a physiotherapy bed and the rectus femoris (m.RF) and biceps femoris (m.BF) were assessed bilaterally according to standardized procedures [23]. In detail, m.RF was measured at 50% of a femur length above the patella [24], whereas m.BF was measured at the midpoint of the line between the fibula head and the ischial tuberosity [25].

#### 2.2.3. Muscle Soreness and Fatigue

To evaluate subjective measures of recovery, participants were asked to rate their perceived lower limb muscle soreness for each leg, where a score of 0 corresponded to “no soreness” and 10 corresponded to “maximal soreness” [11]. In addition, perceived fatigue was also evaluated with a numeric rating scale from 0 (absence of fatigue) to 10 (extremely fatigued) [26]. 

### 2.3. Statistics and Data Analyses

Since no previous study has compared these three recovery strategies, the software G*Power was used to estimate a minimum sample size of 11 participants, based on the effects of CWI on muscle soreness as previously reported [27]. Considering the possible risk of drop-out, we aimed to recruit 16 participants. Since all the subjects completed all the assessments and procedures, a posteriori analysis based on muscle soreness findings reported an achieved power of 0.97. Statistical analyses were performed with SPSS version 23 (IBM, Armonk, NY, USA). This is the primary analysis of these data. Data are reported as the means, standard deviation, counts, and proportions (%) as appropriate. Two-tailed testing was performed. Normality testing using the Shapiro–Wilk test was performed for all datasets, and all parameters were normally distributed. Analysis of variance (ANOVA) was performed considering the within-factor group (3 recovery interventions) and within-factor time (3 time points). In the event of a statistically significant interaction effect, simple main effects were performed. Greenhouse–Geisser correction was applied in case of lack of sphericity. The effect size was determined by _p_η^2^. Significance was set for *p* < 0.05.

## 3. Results

Sixteen male soccer players were recruited and completed the cross-over trial (age 22 years, range 20–27), without any exclusion or dropout. Body mass and height were 72.8 ± 8.7 kg and 1.79 ± 0.06 m, with a BMI of 22.7 ± 2.2 kg/m^2^. Fifteen players reported the right lower limb as the dominant, with a history of playing soccer for 16 ± 2 years at subelite level, and with a training volume of 7.4 ± 2.4 h/week. Since no side interaction effects were found for isometric muscle strength and TMG assessment, data are reported for the dominant limb. 

### 3.1. Isometric Muscle Strength

**Figure 2:**
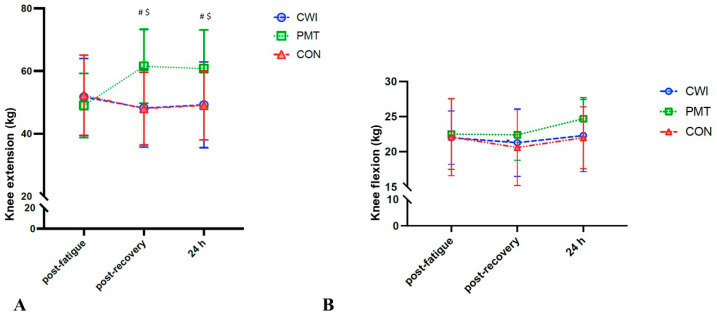
Effects of cold water immersion (CWI, circles, blue), percussive massage therapy (PMT, squares, green), and passive rest (CON, triangles, red) on (**A**) isometric knee extension (kg) and (**B**) isometric knee flexion (kg), post-fatigue protocol, post-recovery intervention, and at 24 h. Time × intervention analysis of variance on 16 participants: # significant difference between PMT and CON; $ significant difference between post-treatment and post-fatigue for PMT. Significance for *p* < 0.05.

Considering IKE, a significant time x recovery interaction was found (F_4,60_ = 6.513, *p* < 0.001, _p_η2 = 0.303). Simple main effects suggested no significant differences between recovery interventions post fatigue, whereas at post-recovery, compared to CON, only PMT resulted in increased strength by 13.6 kg (95% CI: 5.1–22.1) (*p* = 0.004). In addition, at 24 h, compared to CON, only PMT resulted in increased strength by 11.8 kg (95% CI: 3.8–19.8) (*p* = 0.007). No significant interaction effect was reported for IKF (Figure 2). 

### 3.2. TMG

Considering TMG Tc, a significant time × recovery interaction was found in m.RF (F_4,60_ = 4.040, *p*= 0.006, _p_η2 = 0.212), whereas no significant interaction was found in m.BF. Simple main effects suggested no significant differences between recovery interventions post fatigue, whereas at post-recovery, compared to CON, CWI resulted in a longer Tc by 2.7 ms (95% CI: 0.3–5.0) (*p* = 0.027). No significant differences were found at 24 h. Additionally, no significant interaction effect was reported for m.RF Dm, or for m.BF Tc and Dm (Table 1). 

### 3.3. Muscle soreness and Fatigue

**Figure 3:**
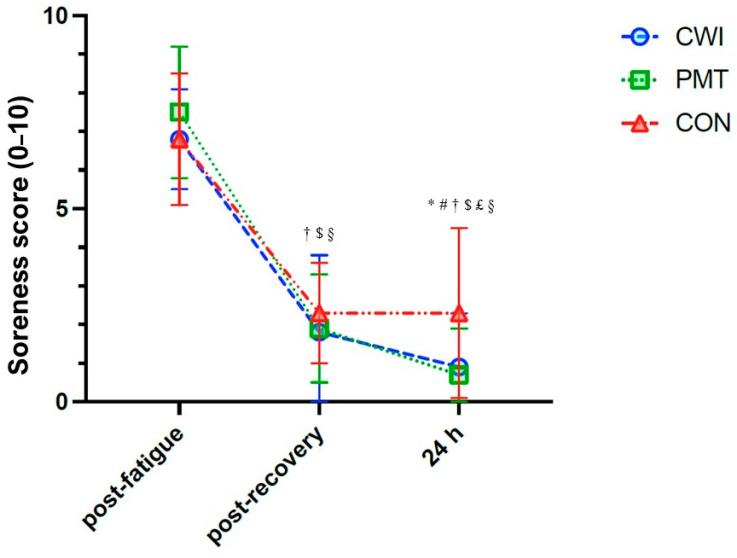
Effects of cold water immersion (CWI, circles, blue), percussive massage therapy (PMT, squares, green), and passive rest (CON, triangles, red) on muscle soreness perception (score 0–10), post-fatigue protocol, post-recovery intervention, and at 24 h. Time x intervention analysis of variance on 16 participants: * significant difference between CWI and CON; # significant difference between PMT and CON; † significant difference between post-treatment and post-fatigue for CWI; $ significant difference between post-treatment and post-fatigue for PMT; £ significant difference between 24 h and post-treatment for PMT; § significant difference between post-treatment and post-fatigue for CON. Significance for *p* < 0.05.

Considering muscle soreness, a significant time × recovery interaction was found (F_4,60_ = 3.095, *p* = 0.022, _p_η2 = 0.171). Simple main effects suggested no significant differences between recovery interventions post fatigue nor post recovery, whereas at 24 h, compared to CON, CWI resulted in −1.5 95%CI: −2.9–−0.2 (*p*= 0.047) and PMT resulted in −1.6 95%CI: −3.0–−0.2 (*p*= 0.026) (Figure 3). For fatigue, a non-significant time × recovery interaction was found.

## 4. Discussion

To the best of our knowledge, this is the first study comparing the effects of CWI, PMT, and a passive resting protocol on different neuromuscular and perception measures of recovery after a fatiguing protocol, with assessments repeated both immediately after the treatment and at 24 h, providing some preliminary evidence of acute and short-term recovery after these interventions. The main findings of this study suggest that both CWI and PMT might induce neuromuscular responses and soreness perception changes post exercise, and acting on different mechanisms and outcomes might be considered for exercise recovery. Indeed, in this cross-over study, we evaluated the effects of CWI and PMT administered immediately after a high-intensity fatiguing protocol, compared to a passive rest condition, on the isometric strength of the thigh muscles, muscle contractile properties, and subjective measures such as muscle soreness and fatigue. 

The isometric strength of the extensor muscles was found to significantly increase immediately after the PMT administration, and it remained at similar values also after 24 h. In contrast, no significant changes were observed in the CWI and CON conditions compared to the post-fatigue assessment, suggesting that both these recovery conditions were insufficient in improving isometric strength. In contrast, no significant changes were found for the isometric strength of the flexor muscles. Conflicting results are present in the literature regarding the effects of percussive therapy on strength improvement; indeed, although current findings could seem to be in line with the results reported in a recent review when compared with no treatment [10], it should be noted that the presented literature seems to be conflicting, with results suggesting no effects on strength [28], or primarily refers to post-injury recovery [29]. Furthermore, a recent repeated-measure, single-group design study found that massage guns could have little effect on physical measures when applied for five minutes immediately following strenuous calf exercise in active young adults [11]. It should be noted that in these reported studies by Konrad et al. and Leabeater et al., PMT was performed on the calf muscle, at 53 Hz for 5 min. Due to the scarcity of the literature and the different protocols that have been applied in terms of condition (at rest or after fatigue), massage protocol (frequency of the stimulation, device, and duration), and assessed muscles, the results should be cautiously considered. In the present study, we applied the PMT bilaterally and on both the anterior and posterior thigh muscles, involved in knee flexion and extension. In addition, to the best of the authors’ knowledge and according to the literature [11], there is a lack of knowledge regarding the effects of acute PMT across a longer time frame of evaluation. In our study, we found that the isometric strength of the thigh muscles could increase at 24 h from the PMT application. Although not directly comparable, other types of massage protocols have been extensively studied. Massage therapy has been suggested to not provide any effect on isometric knee extensor strength after running, whereas vibrating foam rollers might improve physical performance after eccentric lower body exercise, maybe due to an increase in body temperature and blood flow [30]. If little is known about the effects of PMT on muscle strength after strenuous exercise, CWI has been widely investigated, despite suggesting conflicting results [4,27]. Indeed, although CWI could be effective after high-intensity exercise, improving muscular power, muscle soreness, creatine kinase, and perceived recovery 24 h after exercise [4], there is some conflicting evidence in terms of the effects of CWI on the recovery of strength performance compared to passive or other recovery modalities [4,27]. Also, it should be noted that CWI characteristics usually differ in the reported studies, with immersion lasting between 10 and 15 min and water temperature being between 8 and 15 °C. As such, despite an individualized protocol being preferred, in our study, we used CWI conditions in line with those reported in the literature [4].

Tensiomyography has been used as a non-invasive measure of muscle contractile properties, and in particular, the time of contraction and muscle belly displacement have been interpreted as measures of explosive power and stiffness, respectively [22]. In particular, TMG has been hypothesized to assess neuromuscular fatigue in different sports and conditions [19,31]. Compared to other massage interventions (such as manual therapy or foam rolling), PMT was suggested to increase Tc and Dm compared to the post-exercise assessment and compared to the untreated leg [32]. Although, in this study, we did not find any significant change in Tc after PMT, a trend for increased Dm, although not reaching statistical significance, could support the use of PMT to reduce muscle stiffness after exercise. In contrast, results from the literature suggest that CWI after exercise might induce a trend for a transient decrease in Dm after the treatment, and an increase in Tc [33], although no significant changes were reported at 24 and 48 h after training compared with passive resting [34]. Decreased muscle temperature is associated with increased muscle stiffness [35], and water temperature could be the primary responsible mechanism for such alterations, since differences have been previously reported between hot water and cold water immersion [33]. The results from our study are in line with such findings, especially regarding the increase in Tc in the rectus femoris muscle immediately after CWI, but not at 24 h. If the interpretation of the change in Dm is commonly referred to as muscle stiffness alterations, it is still debated whether fatigue recovery is represented by an increase or a decrease in Tc, and therefore this finding should be cautiously interpreted. Interestingly, a consistency was found between the isometric strength and TMG changes observed in the PMT condition, suggesting that the anterior thigh muscles were more affected by the recovery intervention. 

In our study, both PMT and CWI were found to significantly reduce muscle soreness perception immediately after the recovery intervention and at 24 h, without differences between the two protocols and more efficiently compared to the passive resting condition. Such findings are in line with the literature, suggesting that these recovery modalities might provide their major effects on subjective measures of pain and fatigue [4,10,11]. Nevertheless, several authors have suggested that it is not possible to exclude that part of the findings, especially those on subjective measures of recovery, might be influenced by a placebo effect [33]. 

### Limitation and Future Perspectives

This study has some limitations that should be discussed. The training level of the participants categorized them as highly trained soccer players (Tier 2), and different patterns/behaviors might be observed in either recreational or elite athletes. The proposed interventions did not allow for blinding, and it is not possible to exclude a placebo-related bias. Also, subjective scales on pain and muscle soreness might have a limited validity and future studies could use algometers and other objective measures. Although TMG measurements do not rely on voluntary contraction and the protocol was performed identically after each condition, to reduce the risk of bias, the researcher who performed all the assessments was blinded to the recovery intervention the participants performed. The proposed recovery interventions were designed based on some of the most commonly reported protocols in terms of the frequency of stimulation/temperature and the duration of the treatment; due to the variety of protocols present in the scientific literature, the present results should be cautiously compared in the context of the scientific literature. We were not able to provide any measure of muscle temperature or blood flow; therefore, it was not possible to link such physiological responses to the assessed outcomes. The fatiguing protocol was designed based on the literature to provide a high-intensity stimulus mimicking a typical team sports training, to be repeatable in the three experimental sessions, as demonstrated by comparable outcomes immediately after the fatiguing protocol and before the recovery intervention. Finally, since no measurements were performed before the fatiguing protocol, it is not possible to determine whether the reported changes indicated better overall recovery; nonetheless, this study aimed to measure the different effects of these typical recovery strategies on fatigued muscles, and future studies should focus on how these evaluations could be considered as indices of recovery, including other typical assessments including jump and sprint performance. Nevertheless, these findings are encouraging and suggest that the acute application of PMT and CWI are both helpful in reducing perceived muscle soreness. Future studies should be performed considering the above-mentioned limitations, comparing neuromuscular performance with pre-fatiguing values, and combining the two techniques to assess their independent and interaction effects.

## 5. Conclusions

In conclusion, percussive massage therapy and cold water immersion represent two of the most common recovery techniques adopted in different sports to accelerate recovery after exercise. The results from this randomized cross-over study suggest that PMT could enhance the isometric strength of knee extensors after strenuous exercise, and future studies on larger samples could better define its effects on muscle stiffness assessed by tensiomyography. CWI, in contrast, does not affect muscle strength and could acutely increase contraction time in the extensor muscles. Finally, both treatments were found to be more effective than passive resting to reduce muscle soreness, therefore providing a significant subjective improvement in recovery.

## Figures and Tables

**Table 1:** TMG outcomes post fatigue, post recovery, and at 24 h. Data are presented as means ± standard deviations.

*n* = 16	CWI	PMT	CON	Significance
m.RF Tc (ms) post fatigue post recovery 24 h m.RF Dm (mm) post fatigue post recovery 24 h m.BF Tc (ms) post fatigue post recovery 24 h m.BF Dm (mm) post fatigue post recovery 24 h	22.5 ± 4.3 26.3 ± 5.7 ^a,^* 23.9 ± 3.9 ^^^ 7.6 ± 2.5 7.2 ± 2.4 7.9 ± 2.8 23.5 ± 6.7 26.3 ± 9.6 25.6 ± 9.6 3.8 ± 1.7 4.3 ± 1.7 4.0 ± 1.7	23.5 ± 4.2 23.7 ± 4.3 23.2 ± 4.0 7.8 ± 2.6 9.3 ± 2.5 9.0 ± 3.0 24.3 ± 7.9 25.0 ± 7.4 24.0 ± 6.0 4.4 ± 2.5 4.8 ± 2.4 4.4 ± 2.3	23.6 ± 5.1 23.6 ± 4.1 24.3 ± 3.2 8.2 ± 3.6 9.0 ± 3.6 8.5 ± 3.2 25.0 ± 5.7 23.4 ± 2.9 24.2 ± 5.3 3.4 ± 1.3 3.5 ± 1.4 3.5 ± 1.4	Time × Recovery *p* = 0.006 Time × Recovery *p* = 0.108 Time × Recovery *p* = 0.183 Time × Recovery *p* = 0.797

Notes: CWI: cold water immersion; PMT: percussive massage therapy; CON: control condition. m.RF: muscle rectus femoris; m.BF: muscle biceps femoris; Tc: time to contraction; Dm: muscle displacement. Analysis of variance, main effect, or simple main effect was used for recovery at different time points. Post hoc analysis for ^a^ significant difference compared to CON; * significant difference compared to post fatigue; ^ significant difference compared to post recovery; significant differences for *p* < 0.05.

## Data Availability

All data generated or analyzed during this study will be included in the article as Table(s), Figure(s). Any other data requirement can be directed to the corresponding author upon reasonable request due to ethical and institutional regulations. The data are not publicly available due to privacy and ethical restrictions.

## References

[1] Haller N., Hübler E., Stöggl T., Simon P. (2022). Evidence-Based Recovery in Soccer—Low-Effort Approaches for Practitioners. J. Hum. Kinet..

[2] Nédélec M., McCall A., Carling C., Legall F., Berthoin S., Dupont G. (2013). Recovery in Soccer: Part II-Recovery Strategies. Sports Med..

[3] Crowther F., Sealey R., Crowe M., Edwards A., Halson S. (2017). Team Sport Athletes’ Perceptions and Use of Recovery Strategies: A Mixed-Methods Survey Study. BMC Res. Notes.

[4] Moore E., Fuller J.T., Bellenger C.R., Saunders S., Halson S.L., Broatch J.R., Buckley J.D. (2023). Effects of Cold-Water Immersion Compared with Other Recovery Modalities on Athletic Performance Following Acute Strenuous Exercise in Physically Active Participants: A Systematic Review, Meta-Analysis, and Meta-Regression. Sports Med..

[5] Rowsell G.J., Coutts A.J., Reaburn P., Hill-Haas S. (2011). Effect of Post-Match Cold-Water Immersion on Subsequent Match Running Performance in Junior Soccer Players during Tournament Play. J. Sports Sci..

[6] Higgins T.R., Greene D.A., Baker M.K. (2017). Effects of Cold Water Immersion and Contrast Water Therapy for Recovery from Team Sport: A Systematic Review and Meta-Analysis. J. Strength Cond. Res..

[7] Zandvoort C.S., de Zwart J.R., van Keeken B.L., Viroux P.J.F., Tiemessen I.J.H. (2018). A Customised Cold-Water Immersion Protocol Favours One-Size-Fits-All Protocols in Improving Acute Performance Recovery. Eur. J. Sport Sci..

[8] Cullen M.F.L., Casazza G.A., Davis B.A. (2021). Passive Recovery Strategies after Exercise: A Narrative Literature Review of the Current Evidence. Curr. Sports Med. Rep..

[9] Cheatham S.W., Stull K.R., Kolber M.J. (2019). Comparison of a Vibration Roller and a Nonvibration Roller Intervention on Knee Range of Motion and Pressure Pain Threshold: A Randomized Controlled Trial. J. Sport Rehabil..

[10] Sams L., Langdown B.L., Simons J., Vseteckova J. (2023). The Effect of Percussive Therapy on Musculoskeletal Performance and Experiences of Pain: A Systematic Literature Review. Int. J. Sports Phys. Ther..

[11] Leabeater A., Clarke A., James L., Huynh M., Driller M. (2023). Under the Gun: The Effect of Percussive Massage Therapy on Physical and Perceptual Recovery in Active Adults. J. Athl. Train..

[12] Szymczyk P., Węgrzynowicz K., Trybulski R., Spieszny M., Ewertowska P., Wilk M., Krzysztofik M. (2022). Acute Effects of Percussive Massage Treatment on Drop Jump Performance and Achilles Tendon Stiffness. Int. J. Environ. Res. Public Health.

[13] Altarriba-Bartes A., Peña J., Vicens-Bordas J., Milà-Villaroel R., Calleja-González J. (2020). Post-Competition Recovery Strategies in Elite Male Soccer Players. Effects on Performance: A Systematic Review and Meta-Analysis. PLoS ONE.

[14] Brink M.S., Visscher C., Arends S., Zwerver J., Post W.J., Lemmink K.A.P.M. (2010). Monitoring Stress and Recovery: New Insights for the Prevention of Injuries and Illnesses in Elite Youth Soccer Players. Br. J. Sports Med..

[15] McKay A.K.A., Stellingwerff T., Smith E.S., Martin D.T., Mujika I., Goosey-Tolfrey V.L., Sheppard J., Burke L.M. (2022). Defining Training and Performance Caliber: A Participant Classification Framework. Int. J. Sports Physiol. Perform..

[16] Waldén M., Mountjoy M., McCall A., Serner A., Massey A., Tol J.L., Bahr R., D’Hooghe M., Bittencourt N., Della Villa F. (2023). Football-Specific Extension of the IOC Consensus Statement: Methods for Recording and Reporting of Epidemiological Data on Injury and Illness in Sport 2020. Br. J. Sports Med..

[17] Zubac D., Šimunič B., Buoite Stella A., Morrison S.A. (2020). Neuromuscular Performance after Rapid Weight Loss in Olympic-Style Boxers. Eur. J. Sport Sci..

[18] Ros A.G.M., Holm S.E., Fridén C., Heijne A.I.L.M. (2013). Responsiveness of the One-Leg Hop Test and the Square Hop Test to Fatiguing Intermittent Aerobic Work and Subsequent Recovery. J. Strength Cond. Res..

[19] Buoite Stella A., Cargnel A., Raffini A., Mazzari L., Martini M., Ajcevic M., Accardo A., Deodato M., Murena L. (2023). Shoulder Tensiomyography and Isometric Strength in Swimmers before and after a Fatiguing Protocol. J. Athl. Train..

[20] Olds M., McLaine S., Magni N. (2023). Validity and Reliability of the Kinvent Handheld Dynamometer in the Athletic Shoulder Test. J. Sport Rehabil..

[21] McLaine S.J., Ginn K.A., Fell J.W., Bird M.L. (2018). Isometric Shoulder Strength in Young Swimmers. J. Sci. Med. Sport.

[22] García-García O., Cuba-Dorado A., Álvarez-Yates T., Carballo-López J., Iglesias-Caamaño M. (2019). Clinical Utility of Tensiomyography for Muscle Function Analysis in Athletes. Open Access J. Sports Med..

[23] Buoite Stella A., Galimi A., Martini M., Di Lenarda L., Murena L., Deodato M. (2022). Muscle Asymmetries in the Lower Limbs of Male Soccer Players: Preliminary Findings on the Association between Countermovement Jump and Tensiomyography. Sports.

[24] Šimunič B. (2019). Two-Dimensional Spatial Error Distribution of Key Tensiomyographic Parameters. J. Biomech..

[25] Ðorđević S., Rozman S., Zupet P., Dopsaj M., Maffulli N. (2022). Tensiomyography Allows to Discriminate between Injured and Non-Injured Biceps Femoris Muscle. Biology.

[26] Clemente F.M., Rabbani A., Araújo J.P. (2019). Ratings of Perceived Recovery and Exertion in Elite Youth Soccer Players: Interchangeability of 10-Point and 100-Point Scales. Physiol. Behav..

[27] Ingram J., Dawson B., Goodman C., Wallman K., Beilby J. (2009). Effect of Water Immersion Methods on Post-Exercise Recovery from Simulated Team Sport Exercise. J. Sci. Med. Sport.

[28] Konrad A., Glashüttner C., Reiner M.M., Bernsteiner D., Tilp M. (2020). The Acute Effects of a Percussive Massage Treatment with a Hypervolt Device on Plantar Flexor Muscles’ Range of Motion and Performance. J. Sports Sci. Med..

[29] Mansuri U., Patel S. (2021). Effectiveness of Theragun and Ergonomic Advice in Patients with Low Back Pain among Bus Drivers-A Randomized Controlled Trial. Int. J. Sci. Res..

[30] Romero-Moraleda B., González-García J., Cuéllar-Rayo Á., Balsalobre-Fernández C., Muñoz-García D., Morencos E. (2019). Effects of Vibration and Non-Vibration Foam Rolling on Recovery after Exercise with Induced Muscle Damage. J. Sports Sci. Med..

[31] Lohr C., Schmidt T., Medina-Porqueres I., Braumann K.M., Reer R., Porthun J. (2019). Diagnostic Accuracy, Validity, and Reliability of Tensiomyography to Assess Muscle Function and Exercise-Induced Fatigue in Healthy Participants. A Systematic Review with Meta-Analysis. J. Electromyogr. Kinesiol..

[32] García-Sillero M., Benítez-Porres J., García-Romero J., Bonilla D.A., Petro J.L., Vargas-Molina S. (2021). Comparison of Interventional Strategies to Improve Recovery after Eccentric Exercise-Induced Muscle Fatigue. Int. J. Environ. Res. Public Health.

[33] Mur-Gimeno E., Sebio-Garcia R., Solé J., Lleida A., Moras G. (2022). Short-Term Effects of Two Different Recovery Strategies on Muscle Contractile Properties in Healthy Active Men: A Randomised Cross-over Study. J. Sports Sci..

[34] Sánchez-Ureña B., Rojas-Valverde D., Gutiérrez-Vargas R. (2018). Effectiveness of Two Cold Water Immersion Protocols on Neuromuscular Function Recovery: A Tensiomyography Study. Front. Physiol..

[35] Point M., Guilhem G., Hug F., Nordez A., Frey A., Lacourpaille L. (2018). Cryotherapy Induces an Increase in Muscle Stiffness. Scand. J. Med. Sci. Sports.

